# Systemic immune-inflammation index as a prognostic marker in heart failure with non-dialysis chronic kidney disease: a two-cohort derivation and validation study with head-to-head comparison against natriuretic peptides

**DOI:** 10.3389/fcvm.2026.1928233

**Published:** 2026-09-03

**Authors:** Tao Chen, Manyang Chen, Mingzhu Hong, Minghui Zhu, Can Chen

**Affiliations:** 1 Department of Cardiology, Affiliated Hospital of Guangdong Medical University, Jinan University, Guangzhou, China; 2 Department of Cardiology, Affiliated Hospital of Guangdong Medical University, Zhanjiang, Guangdong, China; 3 Department of Cardiology, Affiliated Hospital of Guangdong Medical University, The First Huizhou Affiliated Hospital of Guangdong Medical University, Huizhou, Guangdong, China

**Keywords:** cardiorenal syndrome, chronic kidney disease, heart failure, MIMIC-IV, natriuretic peptides, prognosis, systemic immune-inflammation index

## Abstract

**Introduction:**

Heart failure (HF) and chronic kidney disease (CKD) frequently coexist and share chronic low-grade inflammation, oxidative stress, and thrombotic activation. Natriuretic peptides remain the cornerstone of HF prognostication, but their interpretation is confounded by reduced renal clearance, leading to false elevations and attenuated prognostic specificity in CKD. The systemic immune-inflammation index (SII), derived from routine blood counts, integrates neutrophil-driven inflammation, lymphocyte-mediated immunity, and platelet-associated thrombosis, and may provide a renal-function–independent adjunct to natriuretic peptides among patients with non-dialysis CKD. We aimed to evaluate whether SII provides incremental prognostic information to BNP and NT-proBNP in HF, with particular attention to the non-dialysis cardiorenal spectrum.

**Methods:**

We conducted a two-cohort study. The derivation cohort included 1,284 adults hospitalized for chronic HF at Guangdong Medical University Affiliated Hospital (2018–2023). The external validation cohort comprised adults with a first HF hospitalization from MIMIC-IV v3.1 (2008–2022). After excluding patients without SII data and those with incomplete covariates, 11,767 patients were included in the primary analysis. The systemic immune-inflammation index (SII), a composite inflammatory marker derived from routine complete blood count, was calculated as platelet count (×10^9^/L) × neutrophil count (×10^9^/L) / lymphocyte count (×10^9^/L). Patients were stratified by CKD-EPI estimated glomerular filtration rate (eGFR) into >=60, 30–59, and <30 mL/min/1.73 m^2^. Cox proportional hazards regression, C-index comparisons, net reclassification improvement (NRI), integrated discrimination improvement (IDI), and SII × CKD-stage interaction tests were performed.

**Results:**

SII predicted mortality in the derivation cohort (HR 1.012, 95% CI 1.007–1.017, *P* = 7.5 × 10⁻⁶) and added modest incremental value to NT-proBNP.

**Conclusion:**

SII is an inexpensive, universally available prognostic marker in HF patients with non-dialysis CKD. It does not replace NT-proBNP, but provides a statistically significant yet clinically modest incremental signal in CKD—across the non-dialysis CKD spectrum. SII may serve as a practical adjunct, not a substitute, for risk stratification across the non-dialysis cardiorenal spectrum.

## 1 Introduction

Heart failure (HF) is the end-stage of many cardiovascular diseases and is associated with a 5-year mortality exceeding 50% (1). The growing burden of HF with preserved ejection fraction underscores the need for robust, broadly applicable risk stratification tools (29). Natriuretic peptides, particularly NT-proBNP, are established biomarkers for HF diagnosis and prognosis (4, 5). They reflect myocardial wall stress and neurohormonal activation, and their elevation portends adverse outcomes across the full spectrum of left ventricular ejection fraction (LVEF).

Yet natriuretic peptides have important limitations in the large and growing subgroup of patients with concomitant chronic kidney disease (CKD). HF and CKD coexist in 40%–60% of hospitalized patients and share bidirectional pathophysiological pathways collectively termed the cardiorenal syndrome (2, 3, 30, 31). BNP and NT-proBNP are cleared, at least in part, by renal filtration; as estimated glomerular filtration rate (eGFR) declines, their half-lives prolong and circulating concentrations rise (6–8). Consequently, markedly elevated natriuretic peptide levels in advanced CKD may reflect reduced renal clearance rather than worsening cardiac congestion, reducing their prognostic specificity and complicating clinical interpretation (9, 10). In older adults, renal insufficiency itself predicts cardiovascular events and mortality independently of traditional risk factors (32).

Chronic low-grade inflammation is another shared feature of HF and CKD. Inflammatory cytokines promote cardiomyocyte apoptosis, myocardial fibrosis, endothelial dysfunction, and adverse ventricular remodeling in HF (12, 33, 34). In CKD, systemic inflammation driven by uremia, oxidative stress, gut dysbiosis, and innate immune activation contributes to accelerated atherosclerosis and cardiovascular death (3, 31, 35). Conventional inflammatory markers such as C-reactive protein and interleukin-6 are not universally standardized or consistently available across all clinical settings in clinical practice, whereas blood-cell-derived indices are universally available (13, 14). The systemic immune-inflammation index (SII), calculated as platelet count×neutrophil count/lymphocyte count, integrates neutrophil-driven inflammation, lymphocyte-mediated immunity, and platelet-associated thrombosis into a single routine laboratory variable (11). Unlike natriuretic peptides, SII is not directly dependent on glomerular filtration, suggesting that its prognostic signal may remain more stable as renal function deteriorates. Similar composite inflammatory indices, including the neutrophil-to-lymphocyte ratio (NLR), platelet-to-lymphocyte ratio (PLR), and systemic inflammation response index (SIRI), have also been associated with cardiovascular prognosis (13, 15, 36–38).

Several questions remain unresolved. Most prior SII studies in HF are single-center and lack external validation (39–41). SII has rarely been compared head-to-head with both BNP and NT-proBNP in the same cohort. The influence of renal function on the relative prognostic value of SII vs. natriuretic peptides is incompletely understood, and whether SII retains or gains value across CKD stages has not been rigorously examined in large, well-characterized cohorts. Guideline-directed medical therapy (GDMT)—including angiotensin receptor–neprilysin inhibition, mineralocorticoid receptor antagonists, and sodium–glucose cotransporter-2 inhibitors—modulates neurohormonal and inflammatory pathways in HF (42–46), yet the prognostic value of SII across treatment intensities remains unclear. Finally, whether SII provides incremental prognostic information to natriuretic peptides in CKD-HF patients is of direct clinical relevance.

To address these gaps, we designed a two-cohort study. First, we used a Chinese HF cohort enrolled according to the 2018 Chinese HF guidelines (16) to derive the association between SII and long-term all-cause mortality. Second, we externally validated these findings in MIMIC-IV, a large U.S. intensive care database accessible through PhysioNet (17, 18), and compared SII with BNP and NT-proBNP with particular attention to CKD subgroups. We hypothesized that SII would provide stable prognostic information across the spectrum of renal function and would offer incremental value to natriuretic peptides in patients with CKD. Accordingly, this study aimed to evaluate whether SII provides incremental prognostic information to BNP and NT-proBNP in patients with HF, with particular attention to the non-dialysis cardiorenal spectrum.

## 2 Methods

### 2.1 Study design and populations

#### 2.1.1 Derivation cohort

We retrospectively enrolled consecutive adult patients hospitalized for chronic HF at Guangdong Medical University Affiliated Hospital between August 2018 and January 2023. Inclusion criteria were age ≥18 years and a clinical diagnosis of chronic HF according to the 2018 Chinese guidelines (16). We excluded patients with NYHA class I, acute myocardial infarction within 24 h, in-hospital death, discharge against medical advice, or missing follow-up and key covariate data, mirroring the pragmatic approach used in MIMIC-IV. The study was approved by the hospital ethics committee (PJKT2023-062); informed consent was waived because of the retrospective design.

#### 2.1.2 External validation cohort

MIMIC-IV v3.1 is a publicly available de-identified electronic health record database from Beth Israel Deaconess Medical Center (2008–2022) (17, 18). We included adults with a first HF hospitalization defined by ICD-9 (428.x) or ICD-10 (I50.x) codes and with complete blood counts allowing SII calculation (11). Patients with hospital stay <24 h or missing survival data were excluded. Because MIMIC-IV is de-identified, the requirement for individual consent was waived.

### 2.2 Heart failure phenotyping

In the derivation cohort, LVEF was measured by transthoracic echocardiography. Patients were classified as HFrEF (LVEF ≤40%), HFmrEF (41%–49%), or HFpEF (≥50%) when LVEF was available, following contemporary guideline definitions (47, 48). Because 46.2% of LVEF measurements were missing and missingness was associated with higher mortality, we included an LVEF-missing indicator in the primary Cox model rather than imputing LVEF. Sensitivity analyses were restricted to patients with known LVEF. In MIMIC-IV, LVEF was unavailable for most patients; we therefore classified HF phenotype using discharge ICD-9/10 codes as HFrEF (428.2x/I502.x), HFpEF (428.3x/I503.x), mixed (428.4x/I504.x), or other/unspecified. Because the two cohorts used different phenotyping methods, cross-cohort phenotype comparisons are presented as complementary evidence and all phenotype-stratified analyses are considered exploratory.

### 2.3 Variables and definitions

#### 2.3.1 CKD stratification and cardiorenal analyses

Estimated glomerular filtration rate (eGFR) was calculated using the CKD-EPI equation (19, 20). Patients were classified into three groups: eGFR ≥60, eGFR 30–59, and eGFR <30 mL/min/1.73 m^2^. Within each stratum, we fitted separate Cox models for SII, BNP, and NT-proBNP and compared discrimination using the C-index. We tested for SII × CKD-stage interaction by including product terms between logSII and eGFR category in the fully adjusted model, and assessed incremental value of SII added to NT-proBNP models using IDI and category-free NRI (26). Patients receiving dialysis or continuous renal replacement therapy were examined in sensitivity analyses. Four-category CKD analyses (≥60, 30–59, 15–29, and <15) were also reported in the Supplementary Material.

SII was calculated as platelet count (×10⁹/L) × neutrophil count (×10⁹/L)/lymphocyte count (×10⁹/L) (11). Because the distribution of raw SII was strongly right-skewed in the derivation cohort, SII was winsorized at the 99.5th percentile and analyzed as a continuous variable per 100-unit increase. In MIMIC-IV, SII was analyzed as log-transformed SII to accommodate its wider dynamic range. These different scaling choices reflect the distinct distributions of SII in the two cohorts; hazard ratios for SII are therefore not directly comparable between cohorts. NLR, PLR, SIRI, and WMR (white blood cell count-to-mean platelet volume ratio) were calculated in MIMIC-IV for secondary comparison (13, 15). eGFR was estimated using the CKD-EPI equation. CKD was defined as eGFR <60 mL/min/1.73 m^2^. Because of the retrospective design and reliance on a single hospitalization record, CKD was pragmatically defined by an admission eGFR <60 mL/min/1.73 m2; we acknowledge that the KDIGO definition requires persistence for ≥90 days (see Limitations). In MIMIC-IV, BNP was recorded in ng/mL (equivalent to pg/1,000 mL) and converted to pg/mL by multiplying by 1,000 (6). NT-proBNP was recorded in pg/mL. Guideline-directed medical therapy (GDMT) intensity in MIMIC-IV was summarized as the count of ACEI/ARB/ARNI, beta-blocker, MRA, and SGLT2i prescriptions during hospitalization, categorized as low (0–1 drugs), moderate (2–3), or high (4) (47, 48).

### 2.4 Outcomes

The primary outcome in the derivation cohort was all-cause mortality from enrollment until January 31, 2024; one-year mortality was also analyzed. In MIMIC-IV, outcomes were in-hospital mortality and mortality at 7, 28, and 365 days.

### 2.5 Statistical analysis

For the primary analyses, statistical significance was set at two-sided *P* < 0.05. The primary endpoint was all-cause mortality in the Guangdong derivation cohort and 1-year mortality in MIMIC-IV. Secondary endpoints (in-hospital, 7-day, and 28-day mortality in MIMIC-IV) and all subgroup analyses (CKD stage, LVEF phenotype, GDMT intensity) were predefined but exploratory and were not adjusted for multiple comparisons. Consequently, subgroup *P*-values should be interpreted with caution. All analyses were designed and performed by T.C. and M.Y.C. using established methods implemented in standard statistical software, with independent verification of all code and outputs by M.Z.H. and M.H.Z.; analytical code is available upon request to facilitate replication.

### 2.6 Sensitivity analyses

We performed several sensitivity analyses to test the robustness of the CKD-stratified findings. First, we excluded patients with acute kidney injury (AKI stage ≥2 or any AKI) because AKI can acutely alter both renal function and inflammatory markers (3). Second, we separately analyzed and excluded patients receiving dialysis or continuous renal replacement therapy (CRRT), since these interventions directly affect leukocyte and platelet counts. Third, we repeated the primary CKD stratification using the MDRD equation instead of CKD-EPI to assess whether the eGFR estimation method influenced results (19). Fourth, we restricted the analysis to patients with HFrEF or HFpEF ICD codes (47, 48). Fifth, in the Guangdong cohort, we reported four-category CKD stratification (≥60, 30–59, 15–29, and <15) to assess robustness across the full renal spectrum.

### 2.7 Handling of non-positive SII, missing data, and proportional hazards assumption

Five patients in the derivation cohort had non-positive SII due to negative neutrophil or lymphocyte counts, which are biologically impossible and likely reflect data-entry or unconstrained imputation artifacts; these patients were excluded from all SII analyses, reducing the analysis sample from *N* = 1,284 (332 events) to *N* = 1,279 (331 events).

To assess robustness to missing covariate data, we performed a sensitivity analysis using multiple imputation by chained equations (m = 20) with Rubin rule pooling, implemented in Python with parametric ordinary least-squares conditional models and Bayesian posterior predictive sampling; only log-transformed NT-proBNP required imputation (*n* = 32 with non-positive values).

Proportional hazards assumptions were evaluated with Schoenfeld residual tests. Where violations were detected, we fitted a time-dependent covariate model with log(time) interactions and report the SII hazard ratio from this model.

## 3 Results

We present the main findings in three parts: baseline characteristics, derivation cohort results, and external validation including head-to-head comparisons with natriuretic peptides. Detailed effect estimates are provided in Tables 1–5; the text highlights key clinical and statistical conclusions.

**Table 1:** Baseline characteristics of the derivation cohort by SII quartile.

Variable	Overall (*N* = 1,284)	Q1 (*n* = 321)	Q2 (*n* = 321)	Q3 (*n* = 321)	Q4 (*n* = 321)	*P*-value
Age, years	72.0 (61.0–81.0)	71.0 (61.0–79.0)	70.0 (58.0–80.0)	72.0 (60.0–81.0)	77.0 (66.0–82.0)	<0.001
SII, × 10⁹/L	728.9 (437.3–1,348.5)	296.3 (225.5–372.4)	567.6 (503.2–641.3)	986.8 (842.1–1,130.7)	2,246.1 (1,728.8–3,785.0)	<0.001
Neutrophils, × 10⁹/L	5.2 (3.7–7.1)	3.4 (2.6–4.5)	4.5 (3.6–5.5)	5.8 (4.7–7.0)	8.4 (6.4–11.3)	<0.001
Lymphocytes, × 10⁹/L	1.4 (0.9–1.9)	1.9 (1.5–2.5)	1.6 (1.2–2.0)	1.3 (1.0–1.8)	0.8 (0.5–1.1)	<0.001
Platelets, × 10⁹/L	206.0 (162.0–258.0)	165.0 (135.0–199.0)	197.0 (160.0–248.0)	227.0 (190.0–278.0)	236.0 (189.0–305.0)	<0.001
Hemoglobin, g/L	125.0 (108.0–140.4)	129.0 (113.0–141.0)	128.0 (111.0–145.0)	126.0 (109.0–141.0)	118.0 (100.0–134.0)	<0.001
NT-proBNP, pg/mL	4,098.0 (1,430.5–9,972.8)	2,885.0 (997.5–6,808.0)	2,976.0 (1,096.0–7,618.0)	4,609.0 (1,638.0–10,313.0)	6,637.0 (2,694.0–15,160.0)	<0.001
Serum creatinine, μmol/L	94.0 (75.0–125.0)	88.0 (74.0–112.0)	91.0 (75.0–114.0)	96.0 (76.0–127.0)	108.0 (75.0–156.0)	<0.001
eGFR, mL/min/1.73 m^2^	62.8 (43.5–82.8)	68.2 (50.0–85.6)	67.3 (50.5–84.8)	61.9 (41.7–81.8)	53.7 (32.8–77.7)	<0.001
Albumin, g/L	36.6 (32.9–39.8)	37.6 (34.5–40.7)	37.6 (34.0–40.9)	36.7 (33.1–39.8)	34.2 (30.7–37.8)	<0.001
Sodium, mmol/L	140.0 (137.5–142.0)	141.0 (139.0–142.4)	140.4 (137.9–142.0)	140.5 (138.0–142.7)	138.5 (135.6–141.0)	<0.001
Potassium, mmol/L	4.0 (3.6–4.3)	3.9 (3.6–4.2)	3.9 (3.6–4.3)	3.9 (3.5–4.3)	4.2 (3.8–4.5)	<0.001
Glucose, mmol/L	5.2 (4.5–6.7)	5.0 (4.4–6.0)	5.0 (4.5–6.1)	5.1 (4.5–6.7)	5.8 (4.7–7.6)	<0.001
Total cholesterol, mmol/L	4.2 (3.4–5.0)	4.0 (3.3–5.0)	4.2 (3.4–5.1)	4.5 (3.6–5.2)	4.0 (3.3–4.8)	0.001
Triglycerides, mmol/L	1.0 (0.8–1.5)	1.0 (0.7–1.4)	1.1 (0.8–1.5)	1.1 (0.8–1.7)	1.1 (0.8–1.4)	<0.001
Male sex	764 (59.5)	186 (57.9)	200 (62.3)	183 (57.0)	195 (60.7)	0.493
HF phenotype						0.011
HFrEF	287 (22.4)	81 (25.2)	71 (22.1)	78 (24.3)	57 (17.8)	
HFmrEF	115 (9.0)	32 (10.0)	26 (8.1)	32 (10.0)	25 (7.8)	
HFpEF	289 (22.5)	88 (27.4)	74 (23.1)	58 (18.1)	69 (21.5)	
Unknown EF	593 (46.2)	120 (37.4)	150 (46.7)	153 (47.7)	170 (53.0)	
NYHA class	3 (2–4)	3 (2–3)	3 (2–3)	3 (2–4)	3 (3–4)	0.005
Coronary heart disease	627 (48.8)	139 (43.3)	158 (49.2)	151 (47.0)	179 (55.8)	0.015
Hypertension	627 (48.8)	136 (42.4)	156 (48.6)	181 (56.4)	154 (48.0)	0.005
Diabetes mellitus	373 (29.0)	84 (26.2)	89 (27.7)	98 (30.5)	102 (31.8)	0.382
Atrial fibrillation	443 (34.5)	135 (42.1)	105 (32.7)	103 (32.1)	100 (31.2)	0.012
Heart valve disease	305 (23.8)	74 (23.1)	83 (25.9)	68 (21.2)	80 (24.9)	0.516
ACEI/ARB/ARNI use	827 (64.4)	223 (69.5)	214 (66.7)	211 (65.7)	179 (55.8)	0.002
Beta-blocker use	756 (58.9)	204 (63.6)	193 (60.1)	194 (60.4)	165 (51.4)	0.013
Diuretic use	755 (58.8)	193 (60.1)	177 (55.1)	200 (62.3)	185 (57.6)	0.282
MRA use	883 (68.8)	238 (74.1)	217 (67.6)	231 (72.0)	197 (61.4)	0.003
SGLT2 inhibitor use	332 (25.9)	96 (29.9)	82 (25.5)	87 (27.1)	67 (20.9)	0.066
All-cause death	332 (25.9)	58 (18.1)	65 (20.2)	89 (27.7)	120 (37.4)	<0.001

HF phenotype was classified as HFrEF [left ventricular ejection fraction (LVEF) ≤ 40%], HFmrEF (LVEF 41%–49%), or HFpEF (LVEF ≥50%) when echocardiographic LVEF was available; otherwise patients were categorized as Unknown EF. LVEF data were available in 691 (53.8%) patients. The *p*-value refers to the overall chi-square test for the distribution of HF phenotype across SII quartiles.

Data are median (25th–75th percentile) or *n* (%). *P*-values for continuous variables from Kruskal–Wallis test; for categorical variables from chi-squared test. SII, systemic immune-inflammation index; NYHA, New York Heart Association; eGFR, estimated glomerular filtration rate; MRA, mineralocorticoid receptor antagonist; SGLT2, sodium-glucose cotransporter 2.

**Table 2:** Association between SII (per 100-unit increase) and all-cause mortality in the derivation cohort.

Model	N	Events	HR (95% CI)	*P*-value
Model 1 (unadjusted)	1,279	331	1.018 (1.014–1.023)	<0.001
Model 2 (age, sex)	1,279	331	1.018 (1.013–1.022)	<0.001
Model 3 (+NYHA, LVEF missing)	1,279	331	1.016 (1.012–1.021)	<0.001
Model 4 (+renal and nutritional markers)	1,279	331	1.012 (1.007–1.017)	<0.001
Model 5 (+comorbidities and medications)	1,279	331	1.012 (1.007–1.017)	<0.001
Model 6 (further adjust for logNT-proBNP)	1,247	327	1.012 (1.007–1.017)	7.5 × 10⁻⁶

HR, hazard ratio; CI, confidence interval; EF, ejection fraction; NT-proBNP, N-terminal pro-B-type natriuretic peptide.

**Table 3:** External validation: association between logSII and mortality in MIMIC-IV.

Outcome	N	Events	HR (95% CI)	*P*-value
In-hospital mortality	11,767	1,428	1.312 (1.262–1.365)	<0.001
7-day mortality	11,767	1,597	1.292 (1.247–1.339)	<0.001
28-day mortality	11,767	2,328	1.337 (1.294–1.382)	<0.001
1-year mortality	11,767	4,015	1.154 (1.122–1.186)	<0.001

HR, hazard ratio; CI, confidence interval. All models are fully adjusted (Model 5).

**Table 4:** C-index comparison of SII, BNP, and NT-proBNP by CKD stage in MIMIC-IV.

CKD stage	Marker	N	Events	HR (95% CI)	*P* value	C-index	Delta C-index vs. SII
eGFR >=60	SII	5,746	1,600	1.137 (1.090-1.186)	<0.001	0.694	ref
eGFR >=60	BNP	2,195	744	0.951 (0.906-1.000)	0.048	0.698	+0.004
eGFR >=60	NT-proBNP	1,481	480	1.338 (1.236-1.449)	<0.001	0.689	-0.005
eGFR 30-59	SII	3,855	1,444	1.154 (1.102-1.208)	<0.001	0.663	ref
eGFR 30-59	BNP	1,970	851	1.106 (1.060-1.154)	<0.001	0.675	+0.012
eGFR 30-59	NT-proBNP	1,017	415	1.305 (1.188-1.433)	<0.001	0.673	+0.010
eGFR <30	SII	2,166	971	1.155 (1.087-1.228)	<0.001	0.680	ref
eGFR <30	BNP	1,391	687	1.107 (1.051-1.167)	<0.001	0.687	+0.007
eGFR <30	NT-proBNP	490	238	1.335 (1.173-1.518)	<0.001	0.682	+0.002
Overall	SII	11,767	4,015	1.154 (1.122-1.186)	<0.001	0.684	ref
Overall	BNP	5,556	2,282	1.053 (1.024-1.081)	<0.001	0.683	-0.001
Overall	NT-proBNP	2,988	1,133	1.321 (1.252-1.395)	<0.001	0.685	+0.001

CKD, chronic kidney disease; SII, systemic immune-inflammation index; BNP, B-type natriuretic peptide; NT-proBNP, N-terminal pro-BNP; HR, hazard ratio; CI, confidence interval. *Δ* C-index = C-index(marker)−C-index(SII).

**Table 5:** Association between logSII and 1-year mortality by HF phenotype in MIMIC-IV.

HF phenotype	N	Events	HR (95% CI)	*P*-value
HFrEF	3,748	1,309	1.20 (1.15–1.25)	<0.001
HFpEF	3,291	1,068	1.15 (1.10–1.20)	<0.001
Mixed	1,281	448	1.06 (0.93–1.20)	0.37
Other/unspecified	3,447	1,190	1.15 (1.09–1.21)	<0.001

HF phenotype classified by discharge ICD-9/10 codes. HFrEF, heart failure with reduced ejection fraction; HFpEF, heart failure with preserved ejection fraction. HR, hazard ratio; CI, confidence interval.

### 3.1 Baseline characteristics

**Figure 1:**
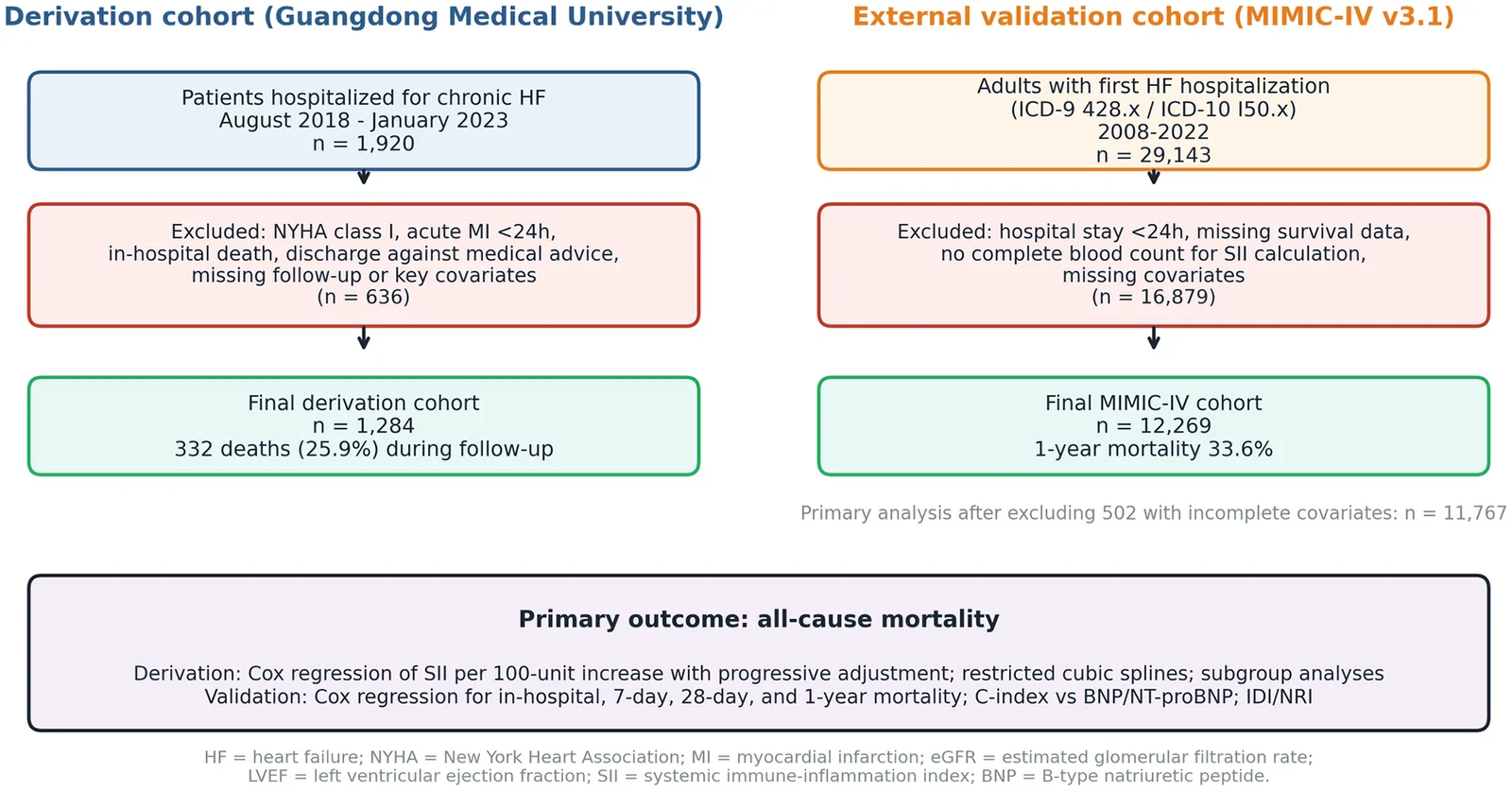
Study flowchart showing the pragmatic, broadly inclusive inclusion criteria applied to both the guangdong derivation cohort and the MIMIC-IV validation cohort..

The derivation cohort included 1,284 patients. Median age was 72 years, 59.5% were male, and 46.2% had missing LVEF. Median SII was 737 (IQR 439-1,367). Patients in the highest SII quartile were older, had more advanced NYHA class, higher NT-proBNP, worse renal function, lower hemoglobin, and higher mortality than those in lower quartiles (Table 1). Figure 1 shows the study flowchart.

In MIMIC-IV, 29,143 patients had a first HF hospitalization, of whom 12,269 (42.1%) had SII data available. After excluding 502 patients with incomplete covariates, 11,767 patients were included in the primary analysis. Median age was 75 years, 54.4% were male, and the 1-year mortality was 33.6%. Patients without SII data differed from those with SII data: they were older, had shorter hospital stays, lower comorbidity burden, and lower short-term and 1-year mortality (Supplementary Table S1). This pattern is consistent with less severely ill patients being transferred or discharged before complete laboratory testing, and supports the robustness of our findings among patients with available SII data while acknowledging that selection bias cannot be fully excluded.

### 3.2 SII and all-cause mortality in the derivation cohort

**Figure 2:**
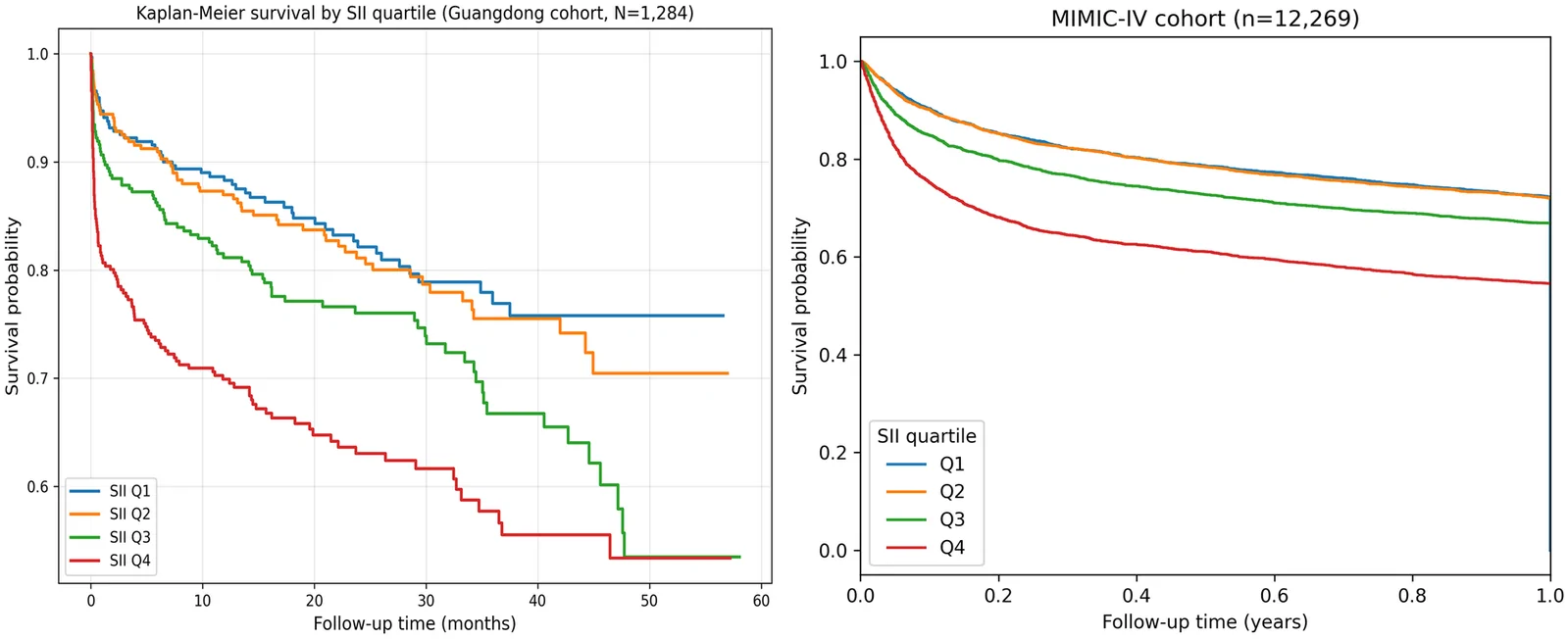
Kaplan–meier survival curves by SII quartile in the derivation and MIMIC-IV cohorts. KM curves based on *n* = 12,269 with SII data; multivariable Cox limited to *n* = 11,767 with complete covariates.

**Figure 3:**
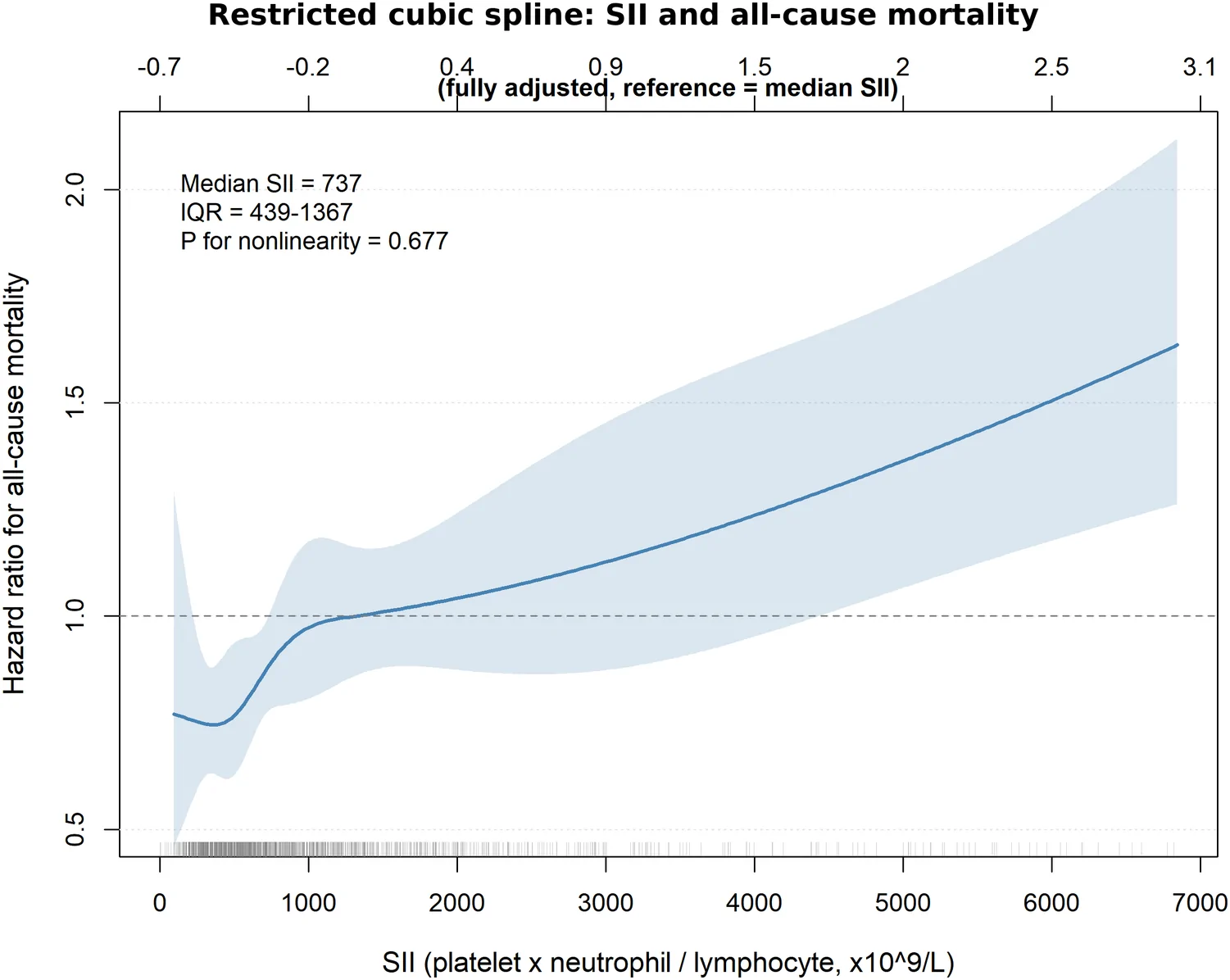
Restricted cubic spline showing dose-response relationship between z-score standardized SII and all-cause mortality in the derivation cohort (fully adjusted model 6). Shaded area represents 95% confidence interval; rug marks show the SII distribution. P for nonlinearity=0.68.

During a median follow-up of 18.4 months, 332 patients (25.9%) died. Kaplan–Meier curves showed a graded decrease in survival across SII quartiles (log-rank *P* < 0.001; Figure 2). When modeled as a continuous variable, each 100-unit increase in SII was associated with higher mortality after progressive adjustment for demographics, NYHA class, LVEF-missing indicator, renal function, nutrition, anemia, comorbidities, GDMT medications, and NT-proBNP (HR 1.012, 95% CI 1.007–1.017, *P* = 7.5 × 10⁻⁶; Table 2). The highest versus lowest SII quartile did not reach statistical significance in the fully adjusted model (HR 1.18, 95% CI 0.61–2.30, *P* = 0.62), suggesting that the prognostic information is captured more continuously across the SII spectrum than by a single extreme-category contrast. Restricted cubic spline analysis after z-score standardization showed a monotonic, approximately linear association between SII and mortality without significant nonlinearity (*P* for nonlinearity=0.68; Figure 3).

In a sensitivity analysis using multiple imputation by chained equations (m = 20) with Rubin rule pooling, the fully adjusted association between SII and mortality was essentially unchanged (HR 1.012, 95% CI 1.007-1.017, *P* = 6.2 × 10⁻⁶), supporting the robustness of the complete-case primary analysis.

### 3.3 External validation in MIMIC-IV

SII was consistently associated with in-hospital, 7-day, 28-day, and 1-year mortality after multivariable adjustment (Table 3). For 1-year mortality, each log-unit increase in SII was associated with a 16% higher risk (HR 1.16, 95% CI 1.12-1.18, *P* < 0.001). Effect sizes were largest for short-term mortality and remained robust across all adjustment models.

### 3.4 SII across LVEF phenotypes

In the derivation cohort, SII remained significant in the LVEF-unknown group (HR 1.011, 95% CI 1.004-1.018, *P* = 0.001) and in the HFrEF subgroup (HR 1.010, 95% CI 1.001-1.019, *P* = 0.039), with trends in HFpEF (HR 1.015, 95% CI 0.999-1.031, *P* = 0.058) and HFmrEF (HR 1.014, 95% CI 0.975-1.055, *P* = 0.48). There was no significant interaction between SII and LVEF-missing status (*P* = 0.95) or continuous LVEF (*P* = 0.53).

**Figure 4:**
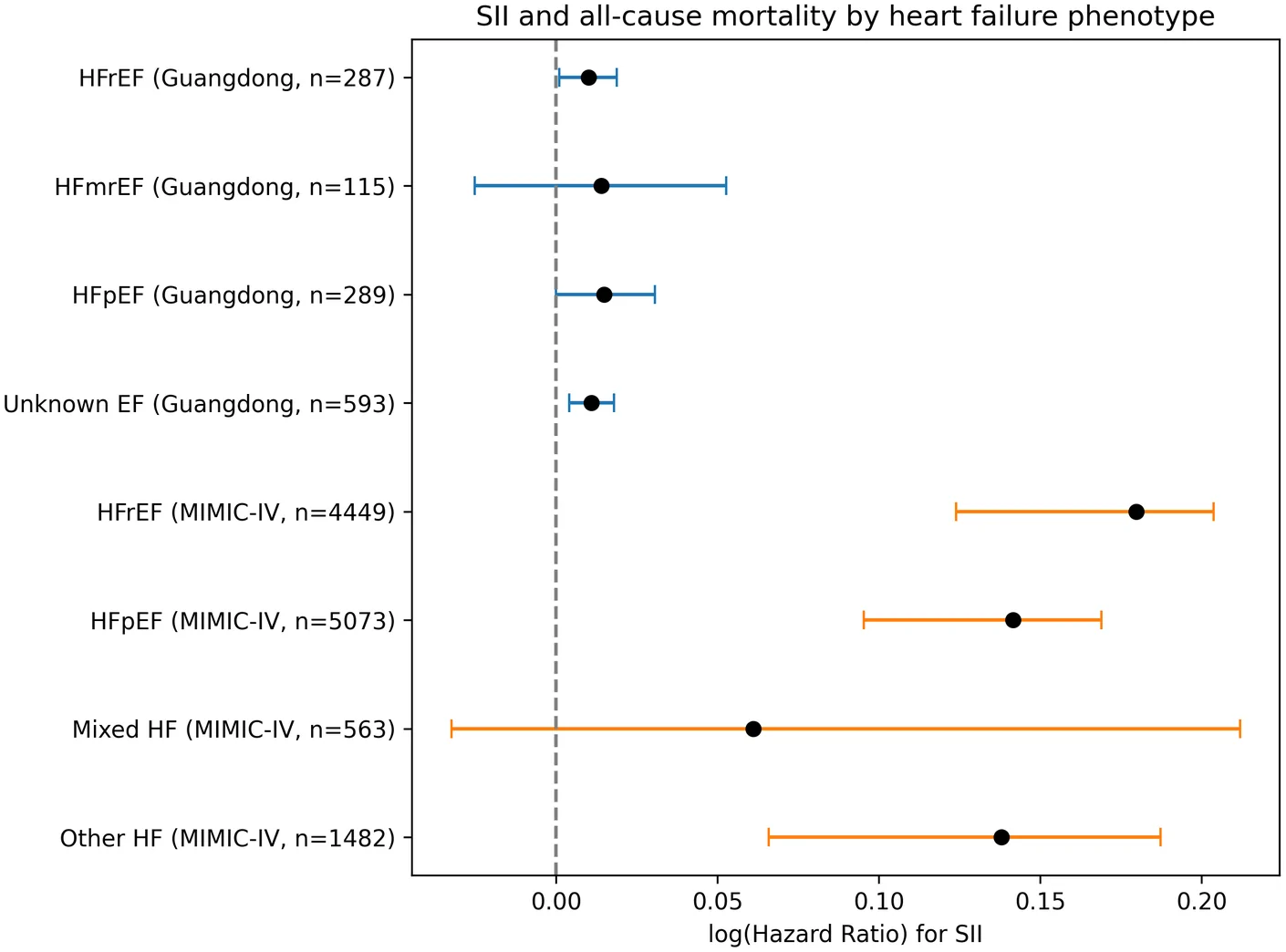
Forest plot of SII association with mortality by HF phenotype in both cohorts. In the Guangdong derivation cohort, hazard ratios correspond to each 100-unit increase in winsorized SII; in MIMIC-IV, hazard ratios correspond to a 1-log-unit increase in log-transformed SII. Because the scales differ, point estimates are not directly comparable between cohorts.

In MIMIC-IV, SII was significantly associated with 1-year mortality in HFrEF (HR 1.20, *P* < 0.001), HFpEF (HR 1.15, *P* < 0.001), and other/unspecified HF (HR 1.15, *P* < 0.001), but not in mixed HF (HR 1.06, *P* = 0.37). Interaction terms between logSII and HFrEF or HFpEF flags were not significant (*P* = 0.35 and *P* = 0.30, respectively; Figure 4). (Table 5).

In sensitivity analyses restricted to patients with known LVEF, SII remained significantly associated with mortality (*n* = 682, 121 events; HR 1.016 per 100-unit increase, 95% CI 1.007–1.025; *P* = 2.87 × 10⁻⁴), consistent with the primary analysis that included the LVEF-missing indicator (Supplementary Table S4).

### 3.5 Head-to-head comparison with natriuretic peptides by CKD stage

**Figure 5:**
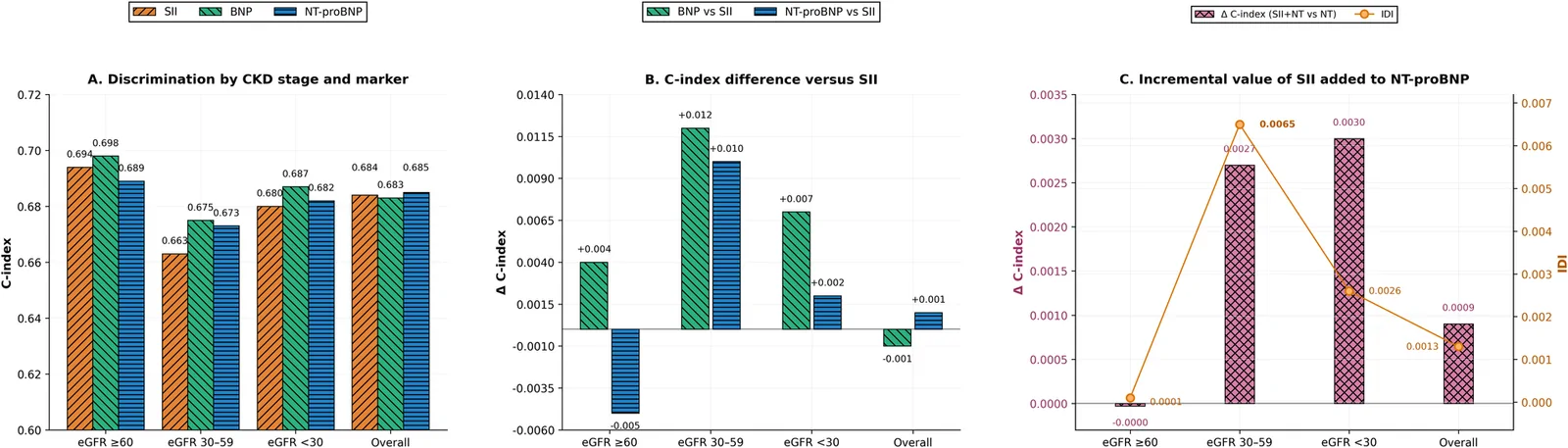
Head-to-head comparison of SII, BNP, and NT-proBNP by CKD stage in MIMIC-IV. **(A)** C-index for each marker within CKD strata. **(B)** Difference in C-index (marker minus SII) versus SII. **(C)** Incremental value of adding SII to NT-proBNP (C-index difference and IDI). Positive *Δ*C-index values indicate higher discrimination for the comparator marker; negative values indicate higher discrimination for SII.

We compared the prognostic performance of SII, BNP, and NT-proBNP across CKD strata in MIMIC-IV using the fully adjusted Model 5 (Table 4). SII remained independently associated with 1-year mortality in every CKD stage: eGFR ≥60 (HR 1.137, 95% CI 1.090–1.186; *P* < 0.001), eGFR 30–59 (HR 1.154, 95% CI 1.102–1.208; *P* < 0.001), and eGFR <30 (HR 1.155, 95% CI 1.087–1.228; *P* < 0.001). The SII × CKD-stage interaction was not statistically significant (likelihood-ratio *χ*^2^ = 0.806, *P* = 0.67), indicating no statistically significant heterogeneity in the SII-mortality association across CKD stages. A non-significant interaction does not prove identical effect sizes; the point estimates appeared similar, but confidence intervals widened in advanced CKD (Figure 5). Kaplan–Meier survival curves stratified by CKD stage are shown in Supplementary Figure S1.

NT-proBNP was the strongest single marker overall, although absolute C-index differences among SII, BNP, and NT-proBNP were small across CKD strata (Table 4). SII remained independently prognostic in every CKD stage, and the SII × CKD-stage interaction was not significant, suggesting a stable renal-function–independent signal. Notably, BNP showed a marginally protective association in the eGFR ≥60 subgroup (HR 0.951, 95% CI 0.906–1.000; *P* = 0.048), which is biologically implausible and likely reflects residual confounding, measurement effects, and model collinearity rather than a true protective effect. We therefore do not interpret this single subgroup finding as evidence for or against BNP prognostic utility and exclude it from the core conclusions; NT-proBNP remains the preferred natriuretic peptide comparator. Thus, SII did not consistently outperform BNP or NT-proBNP in pure discriminative terms; rather, its value lay in providing a stable, renal-function–independent signal that approached the natriuretic peptides even in advanced CKD, where BNP and NT-proBNP interpretation is most confounded by renal clearance.

Adding SII to NT-proBNP yielded statistically significant but clinically small improvements in discrimination in the overall CKD-enriched MIMIC-IV cohort [C-index 0.686 versus 0.685; integrated discrimination improvement [IDI] 0.0013; net reclassification improvement [NRI] 0.175] and in the eGFR <30 subgroup (C-index 0.685 versus 0.682; IDI 0.0026; NRI 0.028). The eGFR 30–59 subgroup showed the largest NRI gain (0.194) with SII added to NT-proBNP, although the absolute C-index increase was small (0.675 versus 0.673). These metrics indicate modest rather than transformative incremental value.

In the Guangdong derivation cohort, SII remained associated with all-cause mortality in the eGFR <30 subgroup (HR 1.013 per 100-unit increase, 95% CI 1.003–1.023; *P* = 0.010) and in the eGFR 30–59 subgroup (HR 1.008, 95% CI 1.001–1.016; *P* = 0.033), with a similar point estimate in the eGFR ≥60 subgroup (HR 1.013, 95% CI 0.999–1.027; *P* = 0.079). In the eGFR <30 subgroup, the C-index was 0.771 for the full SII model and 0.771 for the full NT-proBNP model. Because this subgroup was small (*n* = 165, 78 events), these findings are regarded as supportive rather than confirmatory evidence.

Overall, these results support a complementary role for SII rather than replacement of NT-proBNP. SII provides a stable, renal-function–independent prognostic signal across the non-dialysis cardiorenal spectrum and adds statistically significant but clinically modest incremental value to NT-proBNP, particularly in non-dialysis CKD populations where natriuretic peptide levels are most difficult to interpret.

### 3.6 Sensitivity analyses

After excluding any AKI, the SII effect remained significant although somewhat attenuated (HR 1.130, 95% CI 1.093–1.168; *P* < 0.001). In the dialysis/CRRT subgroup (*n* = 395), SII was not significantly associated with mortality (HR 0.99, *P* = 0.87), whereas NT-proBNP remained significant (HR 1.51, *P* = 0.003), suggesting that dialysis-related hematologic changes may obscure the SII signal. Excluding dialysis/CRRT patients yielded results consistent with the primary analysis (SII HR 1.149, 95% CI 1.116–1.182; *P* < 0.001). Using the MDRD equation instead of CKD-EPI produced nearly identical SII estimates (HR 1.157, 95% CI 1.125–1.189; *P* < 0.001). Four-category CKD analysis showed significant SII associations across all strata, including eGFR <15 (HR 1.178, 95% CI 1.053–1.319; *P* = 0.004). Restriction to HFrEF/HFpEF patients also supported the primary findings (SII HR 1.164, 95% CI 1.129–1.201; *P* < 0.001).

### 3.7 Treatment intensity and selected subgroups

In MIMIC-IV, SII remained prognostic in both low-GDMT (HR 1.15, *P* < 0.001) and moderate-GDMT groups (HR 1.14, *P* < 0.001); the high-GDMT group was too small for reliable estimation. There was a significant negative interaction between SII and GDMT count (*P* = 0.002), suggesting that intensive GDMT may attenuate the excess risk associated with high SII. In age-stratified analysis, SII effects were stronger in patients >75 years.

### 3.8 Proportional hazards sensitivity analysis

Schoenfeld residual testing indicated violations of the proportional hazards assumption for beta-blocker use, diuretic use, and log-transformed NT-proBNP, as well as for SII itself and LVEF-missing status (global *P* < 0.001 under log-time transformation). Fitting a time-dependent covariate model with log(time) interactions for beta-blocker, diuretic, and logNT-proBNP yielded a minimally attenuated SII effect estimate (HR 1.006 per 100-unit increase, 95% CI 1.001-1.011, *P* = 0.020) and significant time-varying coefficients for these three covariates, confirming that the primary SII finding was not sensitive to the specific form of the PH adjustment.

## 4 Discussion

### 4.1 Principal findings

Adding SII to NT-proBNP improved discrimination and reclassification modestly in statistical terms in the overall cohort and in the eGFR <30 subgroup, with absolute C-index gains of 0.001 and 0.003, respectively.

The prognostic association of SII was most evident when modeled continuously rather than as an extreme quartile contrast, consistent with a graded inflammatory-thrombotic risk signal rather than a threshold effect.

### 4.2 Mechanistic interpretation

SII integrates three biologically interconnected axes of the systemic inflammatory response. Neutrophils release proteases and reactive oxygen species that amplify myocardial and vascular injury; lymphopenia reflects immune exhaustion and malnutrition; and activated platelets promote thrombosis and leukocyte recruitment (11, 12, 33). In CKD, each of these axes is amplified by uremic inflammation, oxidative stress, and endothelial dysfunction (3, 31, 35). Unlike NT-proBNP and BNP, whose circulating concentrations are heavily influenced by renal clearance, SII is derived from cellular elements of blood and is not directly dependent on glomerular filtration. This biological distinction likely explains the more stable prognostic signal of SII across eGFR categories and its relative gain in advanced CKD, where natriuretic peptide levels become less specific (6, 9).

### 4.3 Cardiorenal syndrome perspective

The cardiorenal syndrome encompasses a spectrum of bidirectional heart-kidney interactions in which acute or chronic dysfunction of one organ induces dysfunction of the other (2, 3, 30, 31). Regardless of the initiating event, systemic inflammation is a common final pathway (3, 31). The present findings suggest that SII may capture this shared inflammatory-thrombotic burden and thus provide a unifying risk signal across CRS subtypes. Because SII is available immediately from a routine complete blood count, it may be particularly useful in acute CRS, where rapid risk stratification is needed and where natriuretic peptide values may be confounded by acute changes in renal function.

The dialysis/CRRT subgroup (*n* = 395) showed no significant association between SII and mortality (HR 0.99, 95% CI 0.88–1.12; *P* = 0.87), whereas NT-proBNP remained predictive (HR 1.51, 95% CI 1.15–1.97; *P* = 0.003). Hemodialysis and CRRT directly alter leukocyte and platelet counts through membrane activation, anticoagulation, and intermittent clearance; these effects can obscure the inflammatory-thrombotic signal captured by SII. We therefore limit the conclusion that SII is a renal-function–independent prognostic marker to patients with non-dialysis CKD and emphasize that SII is unlikely to be useful in dialysis-dependent populations without further validation.

### 4.4 Comparison with natriuretic peptides

#### 4.4.1 SII across LVEF phenotypes

Of the 691 patients with known LVEF, 682 had complete covariate data and were included in the sensitivity analysis. The prognostic association of SII was consistent across LVEF phenotypes. In the derivation cohort, SII remained significant in the LVEF-unknown group (HR 1.011, 95% CI 1.004–1.018; *P* = 0.001) and in the HFrEF subgroup (HR 1.010, 95% CI 1.001–1.019; *P* = 0.039), with similar trends in HFpEF (HR 1.015, 95% CI 0.999–1.031; *P* = 0.058) and HFmrEF (HR 1.014, 95% CI 0.975–1.055; *P* = 0.48). There was no significant interaction between SII and LVEF-missing status (*P* = 0.95) or continuous LVEF (*P* = 0.53). In MIMIC-IV, SII was significantly associated with 1-year mortality in HFrEF (HR 1.20, *P* < 0.001), HFpEF (HR 1.15, *P* < 0.001), and other/unspecified HF (HR 1.15, *P* < 0.001), but not in mixed HF (HR 1.06, *P* = 0.37). Interaction terms between logSII and HFrEF or HFpEF flags were not significant (*P* = 0.35 and *P* = 0.30, respectively). These findings suggest that the prognostic information carried by SII is largely independent of LVEF category and complementary to phenotype-based risk stratification, extending the HFpEF epidemiology described elsewhere (29).

This consistency has methodological implications. Because 46.2% of the derivation cohort lacked LVEF measurements and missingness was associated with higher mortality, we used an LVEF-missing indicator in the primary Cox model rather than single-value imputation, which can compress variance and bias effect estimates (23, 49). The stable SII effect in LVEF-unknown patients, together with the absence of significant SII × LVEF interactions, supports the interpretation that SII captures a systemic inflammatory-thrombotic component of HF risk that is not fully explained by systolic function. Inflammatory activation is increasingly recognized in both HFrEF and HFpEF, and a marker that retains prognostic value across phenotypes may be particularly useful when echocardiography is unavailable or when patients present with mixed or ill-defined HF categories (47, 48).

LVEF was missing in 46.2% of the derivation cohort, and missingness was associated with higher mortality, raising the possibility of missing-not-at-random (MNAR) bias. Sensitivity analyses in patients with known LVEF (*n* = 682, 121 events; HR 1.016, 95% CI 1.007–1.025; *P* = 2.87 × 10⁻⁴) and in those with missing LVEF (*n* = 565, 206 events; HR 1.010, 95% CI 1.004–1.017; *P* = 0.002) both supported a positive SII-mortality association, suggesting that the primary findings are not driven by the LVEF-missing subgroup (Supplementary Table S4). Nevertheless, the high proportion of missing LVEF remains a limitation.

Our results do not support replacing NT-proBNP with SII. NT-proBNP remained the most discriminative single biomarker overall, consistent with its established role as a marker of myocardial wall stress and hemodynamic congestion (4–6). However, BNP and NT-proBNP are not equivalent in CKD: both are influenced by renal clearance, and their circulating concentrations rise as eGFR falls (7–10). Angiotensin receptor–neprilysin inhibition, while improving outcomes, also alters renal hemodynamics and natriuretic peptide metabolism (50). In the present analysis, SII remained independently prognostic across all CKD stages, the SII × CKD-stage interaction was not significant, and the absolute discrimination of SII approached that of the natriuretic peptides in advanced CKD. These findings suggest that SII is best positioned as a complementary marker—one that is universally available, inexpensive, and relatively robust to renal function—rather than a competitor to NT-proBNP.

### 4.5 Clinical implications

SII offers an immediately available, renal-function–independent risk signal that provides a modest, adjunctive incremental signal to NT-proBNP among patients with non-dialysis CKD.

### 4.6 Strengths and limitations

Strengths include the two-cohort design with external validation, the large sample size of MIMIC-IV, and the head-to-head comparison with both BNP and NT-proBNP across CKD stages. Limitations are those of retrospective observational studies: residual confounding cannot be excluded, SII was measured once at admission, and causal inferences are not justified. LVEF phenotyping differed between cohorts. LVEF was missing in 46.2% of patients; the missing-indicator method was used, and sensitivity analyses in the known-LVEF subset and MICE imputation yielded consistent results. Both cohorts are effectively single-center: the Guangdong cohort represents one Chinese hospital and MIMIC-IV represents one U.S. center. Consequently, differences in healthcare systems, ethnic composition, and clinical practice patterns between China and the United States limit direct extrapolation to other populations or settings. Both cohorts used broadly inclusive, pragmatic inclusion criteria, which improves internal validity but does not remove these generalizability constraints. The high proportion of patients without SII data in MIMIC-IV (57.9%) reflects informative missingness—less severely ill patients were often discharged or transferred before complete blood counts—so the analyzed sample represents a higher-risk, more completely evaluated subgroup rather than an unselected HF population. The Guangdong CKD subgroup with eGFR <30 was relatively small (*n* = 165, 78 events), limiting precision; these analyses should be interpreted as supportive rather than confirmatory. CKD definition was based on a single eGFR measurement and may not fully satisfy the KDIGO 90-day persistence criterion. We did not measure high-sensitivity C-reactive protein or interleukin-6 and therefore cannot directly compare SII with these established inflammatory markers (34, 35). Multiple imputation supported the robustness of the primary complete-case analysis (49). Finally, dialysis and CRRT can alter leukocyte and platelet counts; although we performed sensitivity analyses, the interaction between dialysis status and SII interpretation warrants further study. Our conclusions are restricted to non-dialysis CKD-HF and should not be generalized to dialysis-dependent or acute-only HF populations without further validation. Five patients had non-positive SII due to negative neutrophil or lymphocyte counts (likely data-entry or imputation artifacts) and were excluded from all SII analyses; this did not alter the study conclusions.

One unexpected finding was the apparently protective association between BNP and mortality in the eGFR ≥60 mL/min/1.73 m^2^ subgroup (adjusted HR 0.951, 95% CI 0.906–1.000, *P* = 0.048), which is biologically implausible and should not be interpreted as evidence that BNP is truly protective in patients with preserved renal function. We attribute this observation to a combination of informative measurement, residual confounding, and model collinearity rather than to a causal benefit of BNP (6, 10). Several lines of evidence support this interpretation. First, BNP was available in only 2,195 of 5,746 patients in the eGFR ≥60 stratum, and those tested were not a random sample: they differed in age, comorbidity burden, and clinical indication from patients without BNP measurements, introducing selection bias. Second, stepwise adjustment showed that the BNP coefficient moved from strongly protective (HR ≈0.87) after age and sex adjustment toward the null (HR ≈0.95) once the full set of covariates was included, suggesting that the initial association was driven by unmeasured or partially adjusted prognostic factors rather than by BNP itself. Third, eGFR exhibited a high variance inflation factor (VIF ≈16.8) in this subgroup, indicating substantial multicollinearity among renal, hemodynamic, and neurohormonal variables that can destabilize coefficient estimates. Fourth, sensitivity analyses that excluded patients with AKI or dialysis shifted the overall BNP association back toward the null or weakly positive range (HR 1.02–1.05), reinforcing the view that the eGFR ≥60 finding is not robust. Finally, the magnitude of the apparent protective effect was small and the confidence interval included 1.0, so even if one accepted the point estimate at face value its clinical relevance would be questionable. For these reasons, we do not draw any conclusion about BNP prognostic performance in patients with preserved renal function from this subgroup result alone. Instead, we regard NT-proBNP as the preferred natriuretic peptide comparator throughout the non-dialysis CKD spectrum and present BNP findings as supplementary sensitivity analyses.

Both cohorts used pragmatic, broadly inclusive inclusion criteria, enhancing generalizability. Because eGFR <15 mL/min/1.73 m^2^ was uncommon in the Guangdong cohort, four-category CKD analyses (≥60, 30–59, 15–29, <15) are reported in the Supplementary Material to demonstrate robustness across the full renal spectrum.

## 5 Conclusion

SII is an independent prognostic marker for mortality in HF patients with non-dialysis CKD. It does not replace NT-proBNP overall, but it provides a statistically significant yet clinically modest incremental prognostic signal across CKD stages. In advanced CKD, SII showed the greatest parity with natriuretic peptide discrimination—where natriuretic peptide interpretation is most challenging. Its low cost, routine availability, and stability across the non-dialysis cardiorenal spectrum make it an attractive adjunct, rather than a substitute, for risk stratification in HF patients with renal dysfunction.

## Data Availability

The MIMIC-IV data analyzed in this study are publicly available via PhysioNet (https://physionet.org/content/mimiciv/3.1/). The Guangdong derivation cohort data are available from the corresponding author upon reasonable request, subject to institutional ethics approval.
